# Electrophysiological correlates of episodic migraine chronification: evidence for thalamic involvement

**DOI:** 10.1186/1129-2377-14-76

**Published:** 2013-09-09

**Authors:** Gianluca Coppola, Elisa Iacovelli, Martina Bracaglia, Mariano Serrao, Cherubino Di Lorenzo, Francesco Pierelli

**Affiliations:** 1 G.B. Bietti Foundation IRCCS, Department of Neurophysiology of Vision and Neurophthalmology G.B. Bietti Foundation-IRCCS, Via Livenza 3, Rome 00198, Italy; 2 Department of Medico-Surgical Sciences and Biotechnologies, “Sapienza” University of Rome Polo Pontino, Latina, Italy; 3 Don Carlo Gnocchi Onlus Foundation, Milan, Italy; 4 IRCCS-Neuromed, Pozzilli, IS, Italy

**Keywords:** Chronic migraine, Thalamus, Somatosensory evoked potentials, Central sensitization, Habituation

## Abstract

**Background:**

Episodic migraine is characterized by decreased high-frequency somatosensory oscillations (HFOs), reflecting thalamo-cortical activity, and deficient habituation of low-frequency (LF-) somatosensory evoked potentials (SSEPs) to repetitive sensory stimulation between attacks. Here, we study conventional LF-SSEPs and HFOs in episodic migraineurs who developed chronic migraine (CM).

**Methods:**

Thirty-four episodic (15 interictally [MOii], 19 ictally [MOi]) and 19 CM patients underwent right median nerve SSEPs. The patient groups were compared to a group of 20 healthy volunteers (HV) of comparable age and gender distribution. We measured the N20-P25 LF-SSEP 1st amplitude block and habituation, and, after applying a band-pass filter (450–750 Hz), maximal peak-to-peak latency and the amplitudes of the early and late HFOs.

**Results:**

Reduced early HFOs, lower 1st block LF-SSEPs and deficient habituation characterize MOii. Initially higher SSEP amplitudes and late normal habituation characterize both CM and MOi patients. After the digital filtration, both patient groups showed shortened latency peaks and normalization of early HFO amplitudes with increased late HFOs. When data of MO and CM patients were combined, the monthly number of days with headache negatively correlated with the LF-SSEP slope (r = −0.385, p = 0.006), which in turn negatively correlated with the 1st amplitude block (r = 0.568, p < 0.001).

**Conclusions:**

Our results show abnormalities in chronic migraine that are also reported during attacks in episodic migraineurs, namely early response sensitization and late habituation. The HFO analysis suggests that this sensory sensitization may be explained by an increase in the strength of the connections between the thalamus and cortex compared to episodic migraine between attacks. Whether this electro-functional behaviour is primary or secondary to daily headache, thus reflecting an electrophysiological fingerprint of the somatosensory system central sensitization process, remains to be determined.

## Background

Migraine is one of the most prevalent and disabling neurological disorders [1,2]. It is characterized by recurrent attacks of headache widely variable in duration and intensity, usually accompanied by nausea/vomiting and/or photo-/phono- phobia. In some cases, migraine patients experience a progressive increase in the frequency of the attacks, leading to headache chronification. This clinical condition is defined as 15 or more headache days with 8 or more migraine attacks per month [3]. The exact pathophysiological mechanisms underlying chronic migraine are still under intense scrutiny. Possible culprits for pain chronification include central sensitization and defective central pain control systems [4,5].

During the last decades, clinical neurophysiology methods have allowed in vivo measurements of the migraineur’s electrocortical responses to various sensory stimuli. Altered thalamo-cortical connections [6,7], with cortical dysexcitability [8] and lack of habituation in response to various sensory stimuli, characterize episodic migraineurs’ brains [9]. This abnormal information processing increases during the pain-free days, reaching its maximum just before the attack onset, and disappears in the ictal phase [6,10-16]. Less is known about how mechanisms underlying headache chronification alter this electro-functional profile in episodic patients experiencing a conversion to CM [17].

Evidence has been recently found in favour of persistent somatosensory system central sensitization in chronic headache secondary to medication overuse (medication overuse headache, MOH) by recording cortical somatosensory evoked potentials (SSEPs) [10,18]. To the best of our knowledge, no study has investigated simultaneously SSEP habituation as well as thalamo-cortical connections in episodic migraine patients who developed chronic migraine without the confounding factor of medication overuse. Having this information may contribute to shed more light on the mechanisms underlying headache transformation.

With this specific purpose, we designed the present study to explore whether the sensory cortical response pattern differs between CM patients and patients with episodic migraine without aura recorded both during and between attacks. To do so we recorded low-frequency (LF) SSEP in order to assess habituation/sensitization phenomena. Thereafter, we studied high-frequency oscillations (HFOs) embedded in the common SSEPs in order to identify two bursts of HFOs: an early component thought to be generated by pre-synaptic thalamo-cortical afferents, and a late component reflecting post-synaptic cortical activation [19-21]. We also sought possible correlations between the electrophysiological pattern and clinical features including the number of days with headache and the duration of the chronification phase.

## Methods

Subjects - Among consecutive patients attending our headache clinic, 53 patients gave informed consent to participate in the study (Table 1) which was approved by the local ethics committee. According to the new ICHD-III criteria [3], 19 patients were diagnosed as having chronic migraine (CM) during their first visit. These patients were not affected by medication overuse since the mean monthly tablet intake was 2.7 ± 3.0. Before progressing to CM, all patients had a clear-cut history of episodic migraine without aura (MO, ICHD-II code 1.1). With the exception of 2 patients who had a mild headache (mean VAS = 3), all CM patients underwent the SSEP recordings in a pain-free state. The 2 patients who had a headache had no associated migrainous features. Thirty-four patients were diagnosed as having episodic migraine without aura (ICHD-II code 1.1). Of these, 15 were recorded during the interictal period (MOii), i.e. at least three days before and after an attack, and 19 within a time range of 12 hours before or after the beginning of an attack, then considered as ictal (MOi). Neither chronic nor episodic patients were allowed to take any prophylactic medication in the three months before the recording session. For those patients experiencing a migraine attack, no acute anti-migraine drugs were allowed until the end of the recording session. For comparison, we enrolled 20 healthy volunteers (HV) of comparable age and sex distribution; they had no personal or familial history (1st or 2nd degree relatives) of migraine or any detectable medical conditions. To avoid variability due to hormonal changes, women were recorded outside their pre-menstrual or menstrual periods. All participants received a complete description of the study and granted written informed consent. The project was approved by the ethical review board of the Faculty of Medicine, University of Rome, Italy.

**Table 1:** Demographic data and headache profiles of patients

	**HV (n = 20)**	**MOii (n = 15)**	**MOi (n = 19)**	**CM (n = 19)**
Women (n)	13	12	13	14
Age (years)	38 ± 10	32 ± 7	33 ± 11	33 ± 14
Duration of history of migraine (years)		18.0 ± 12.7	17.3 ± 13.8	17.3 ± 13.8
Days with headache/month (n)		2.0 ± 1.3	3.7 ± 2.5	25.6 ± 6.2
Severity of headache attacks (0–10)		6.8 ± 0.8	8.0 ± 0.9	7.1 ± 0.9
Duration of the chronic headache (month)				15.2 ± 24.0
Tablet intake/month (n)		1.8 ± 2.1	3.2 ± 2.8	2.5 ± 3.0

Data expressed as mean ± SD. HV healthy volunteers; MOii episodic migraneurs without aura studied interictally; MOi episodic migraneurs without aura studied ictally; CM chronic migraine patients; N number of subjects.

### Data acquisition

Somatosensory evoked potentials (SSEPs) were elicited by electrical stimulation applied to the right median nerve at the wrist using a constant current square wave pulse (0.1 ms width, cathode proximal), with a stimulus intensity set at 1.5 times the motor threshold, and a repetition rate of 4.4 Hz. The active electrodes were placed over the contralateral parietal area (C3′, 2 cm posterior to C3 in the International 10–20 system) and on the fifth cervical spinous process (Cv5), both referenced to Fz; the ground electrode was on the right arm [10]. SSEP signals were amplified with a Digitimer™ D360 pre-amplifier (Digitimer Ltd, UK) (band-pass 0.05-2500 Hz, Gain 1000) and recorded with a CED™ power1401 device (Cambridge Electronic Design Ltd, Cambridge, UK).

Subjects sat relaxed in a comfortable chair in a well-lit room with eyes open. They were asked to fix their attention on the stimulus-induced thumb movement. During continuous median-nerve stimulation at the wrist, we collected 500 sweeps of 50 ms, sampled at 5000 Hz. All recordings were acquired and processed using the Signal™ software package, version 4.10 (CED Ltd). Artefacts were automatically rejected using the Signal™ artefact rejection tool if the signal amplitude exceeded 90% of analog-to-digital converter (ADC) range and controlled by visual inspection. This approach we made sure to exclude all severe artefacts but not to remove any signal systematically because background EEG amplitudes are larger in some subjects than in others. The EP-signal was corrected off-line for DC-drifts, eye movements and blinks. Five hundred artefact-free evoked responses recorded in each subject were averaged (“grand average”).

#### Low-frequency SSEPs

After digital filtering of the signal between 0–450 Hz, the various SSEP components (N13, N20, P25 and N33) were identified according to their respective latencies. We measured peak-to-peak amplitudes of the cervical N13 component (recorded under the active Cv5 electrode), and the cortical N20-P25 and P25-N33 components (recorded under the active C3′ scalp electrode).

**Figure 1:**
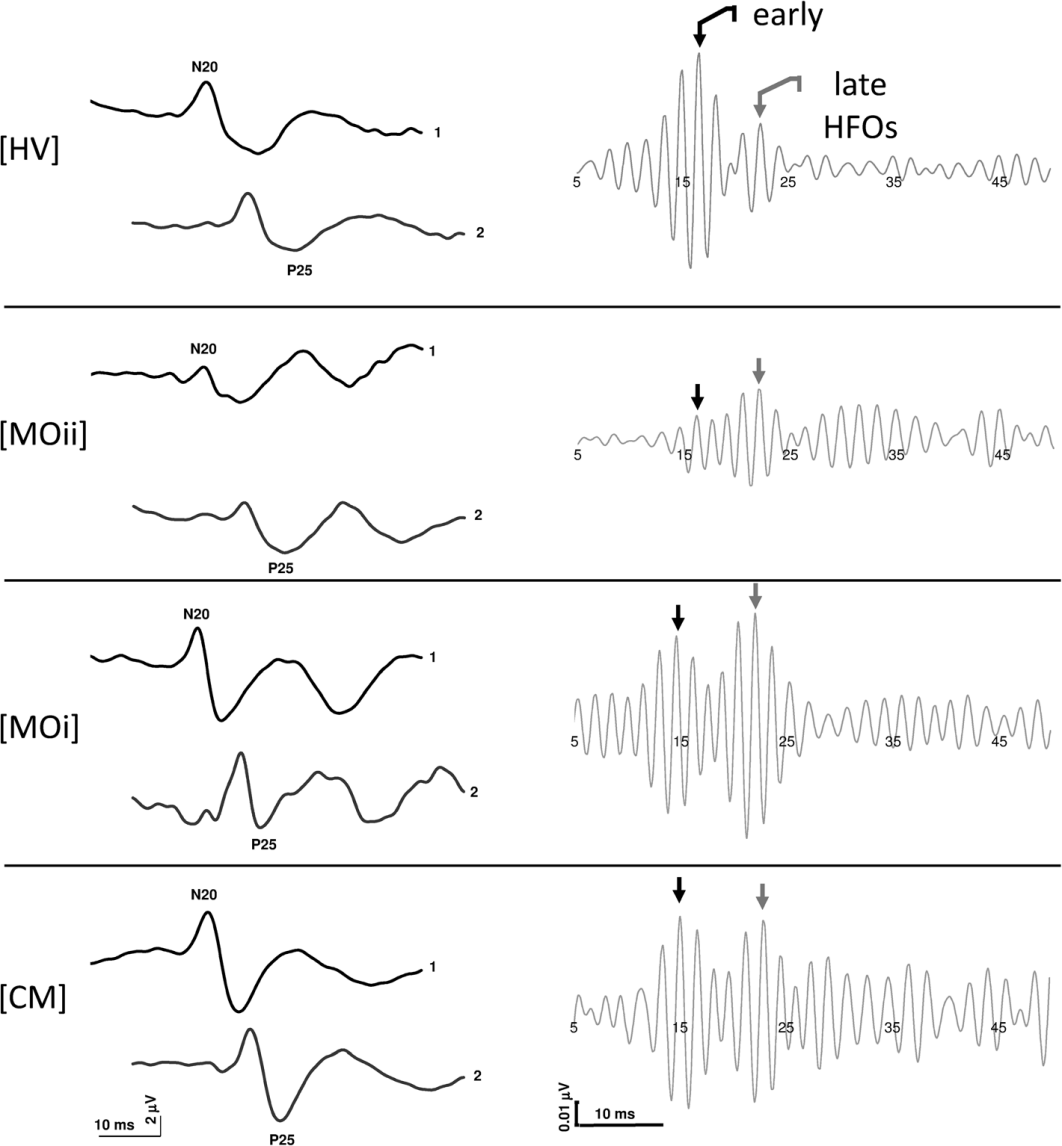
**Schematic representation of the changes in somatosensory evoked potentials (SSEPs) N20-P25 amplitude habituation (left panel) and early and late high-frequency oscillations (HFOs) (right panel) in patients in comparison to the responses obtained from a healthy volunteer.** (HV, healthy volunteer; MOii, migraine without aura between attacks; MOi, migraine during the attack, CM, chronic migraine).

Thereafter, the first 200 evoked responses were partitioned in 2 sequential blocks of 100 responses (Figure 1). Each block was averaged off-line (“block averages”) and analyzed for N20-P25 amplitudes. Sensitization was defined as an increased N20-P25 amplitude recorded during block 1 (after a low number of 100 stimuli), whereas habituation was expressed as the change in N20-P25 amplitude in block 2 compared to block 1 (over a high number of 200 repetitive stimuli) [10].

#### High-frequency oscillations (HFOs)

According to the method described elsewhere [6,22], digital zero-phase shift band-pass filtering between 450 and 750 Hz (Barlett-Hanning window, 51 filter coefficients) was applied off-line in order to extract the HFOs embedded in the parietal N20 SSEP component. In the majority of the recorded traces we were able to identify two separate bursts of HFOs: an early burst occurred in the latency interval of the ascending slope of the conventional N20 component and a late burst in the time interval of the descending slope of N20, sometimes extending into the ascending slope of the N33 peak. In general, the frequency of the oscillations was higher in the first than in the second HFO burst and in between the early and late bursts there was a clear frequency and amplitude decrease, which allowed the two bursts to be separated. In recordings in which a clear distinction between the two components was not possible, we considered HFOs occurring before the N20 peak as early burst and those after the N20 peak as late burst.

After eliminating the stimulus artefact, we measured the latency of the negative oscillatory maximum and the maximum peak-to-peak amplitude separately on the two HFO bursts.

#### Statistical methods

We used the Statistical Package for the Social Sciences (SPSS) for Windows, version 19.0 for all analyses. For grand average SSEPs, component amplitudes were tested in a one-way analysis of variance (ANOVA) with group factor “subjects” (CM patients, MOii and MOi patients, healthy volunteers). To assess changes in SSEP amplitude between blocks 1 and 2, SSEP N20-P25 amplitudes were tested with a repeated-measure ANOVA with group factor “subjects”. Tukey’s test was used for post hoc analyses. Pearson’s correlation coefficient was calculated to test correlations between SSEP amplitudes or habituation and clinical data (disease duration, days with headache, duration of chronic headache). P values of less than 0.05 were considered to indicate statistical significance.

## Results

Assessable SSEP recordings were obtained from all patients and controls participating in the study (explicative traces in Figure 1).

### Low-frequency (LF-) SSEPs

On grand average SSEP recordings after electrical median nerve stimulation, latencies of N13, N20, P25 and N33 components were not different between groups (for each measure F_(3,69)_, p > 0.05).

**Figure 2:**
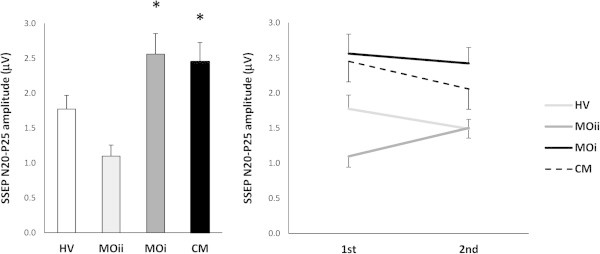
**Somatosensory evoked potential (SSEP) 1st amplitude block average (left panel) and habituation (right panel) in each group (data expressed as mean ± SEM).** HV, healthy volunteer; MOii, migraine without aura between attacks; MOi, migraine during the attack, CM, chronic migraine. (*P < 0.05 vs. HV).

ANOVA testing SSEP amplitude block averages revealed a main effect for factors group (F_(3,69)_ = 6.08, p < 001) and a significant interaction of group by block (F_(3,69)_ = 4.94, p = 0.003). Post hoc analysis showed that the 1st block N20-P25 amplitude was higher in patients with CM (p = 0.04) and MOi (p = 0.02) and tended to be lower in those with MOii (p = 0.06) when compared with HV (Figure 2). Besides, the 1st N20-P25 amplitude block was higher in CM than in MOii patients (p < 0.001). In HV, MOi, and CM patients, N20-P25 amplitude decreased from block 1 to block 2, i.e. habituated (slope −0.28 in HV, -0.13 in MOi, -0.39 in CM, p = 0.594), while in patients with MOii it increased, i.e. did not habituate (+0.40, p = 0.002 vs HV, p = 0.01 vs MOi, p = 0.001 vs MOii, Figure 2).

Only when patients with episodic and chronic migraine were pooled together, the correlation test revealed that monthly days with headache correlated negatively (r = −0.366, p = 0.01) with the amplitude slope of LF-SSEP N20-P25, a measure of habituation. The N20-P25 1st amplitude block correlated negatively with the linear regression slope (r = −549, p < 0.001). The visual analogue scale score did not correlate with any of the neurophysiological parameters.

### High-frequency oscillations (HFOs)

**Figure 3:**
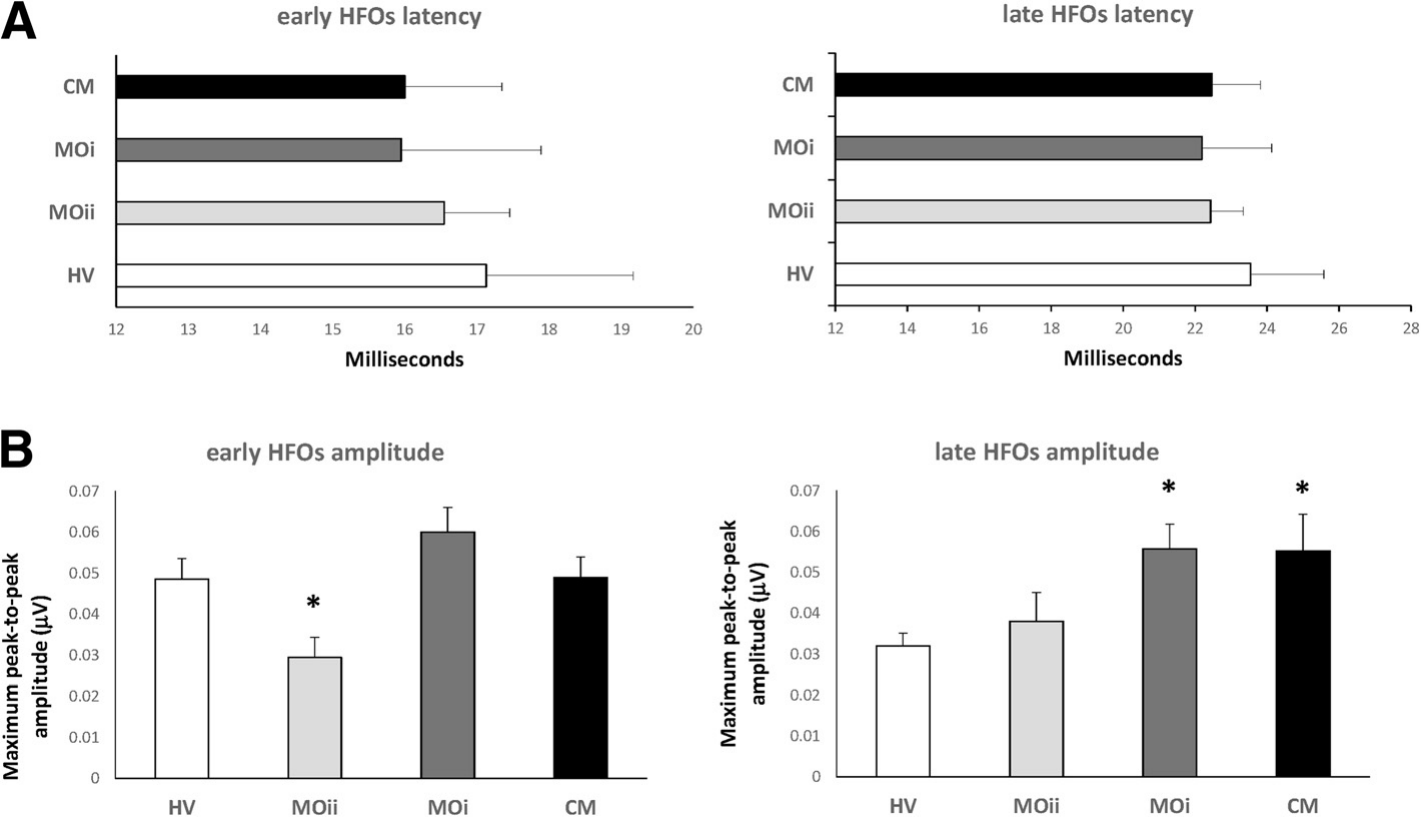
**High-frequency oscillations (HFOs) latency of the negative oscillatory maximum (A) and maximum peak-to-peak amplitude (B), separately on the early (left panel) and late (right panel) HFO bursts (data expressed as mean ± SEM).** (*P < 0.05 vs. HV).

Maximum peak-to-peak amplitudes of the early HFO burst differed between groups (F_(3,69)_ = 3.96, p = 0.01). Post hoc analysis revealed that in MOii patients maximum peak-to-peak amplitudes of the early HFO burst were significantly lower than in HV (p = 0.03), MOi (p = 0.001), and CM (p = 0.02) patients (Figure 3).

Maximum peak-to-peak amplitudes of the late HFO burst differed between groups (F_(3,69)_ = 3.26, p = 0.02). Post hoc analysis showed that in CM and MOi patients maximum peak-to-peak amplitudes of the late HFO burst were significantly higher than in HV (p = 0.014 and p = 0.013 respectively) and tended to be higher than in MOii patients (both p = 0.08) (Figure 3).

Latency of the negative oscillatory maximum of both early and late HFOs did not differ between groups (F_(3,69)_ = 2.13, p = 0.10; F_(3,69)_ = 1.43, p = 0.23 respectively).

When subject groups were considered separately, only in CM and MOi patients was there a positive correlation between early and late HFO maximum peak-to-peak amplitudes (r = 0.584, p = 0.009; r = 0.526, p = 0.02 respectively). Nonetheless, this positive correlation was still present when all subject groups (HV, MOii, MOi, CM) were combined (r = 0.546, p < 0.001), while early and late HFO maximum peak-to-peak amplitudes also positively correlated with the BB-SSEP N20-P25 1st amplitude block (r = 0.359, p = 0.002 and r = 0.299, p = 0.01 respectively).

## Discussion

We designed this study to investigate how headache chronification alters subcortico/cortical somatosensory response patterns of episodic migraine patients experiencing progression to chronic migraine without medication overuse.

In our episodic migraineurs between attacks, we have confirmed previous results showing, on the one hand, that the LF-SSEP N20-P25 amplitude lacks habituation during stimulus repetition despite an initially low response amplitude [10,22], and that this comes in parallel to a reduced somatosensory thalamocortical activity [6,23], as reflected by the low amplitude of the early HFO, on the other hand.

Chronic migraine patients have shown a neurophysiological pattern quite similar to that of episodic migraineurs recorded during an attack. In fact, both groups of patients were characterized by higher amplitudes after low numbers of median nerve electrical stimuli (block 1), reflecting sensory cortex sensitization, and by response habituation over sequential block averages. This combination of subcortico/cortical electrophysiological patterns observed in our chronic migraineurs was previously defined as a condition of “never-ending migraine attack” [22]. Moreover, after band-pass filtering, in both CM and MOi patients the interictal episodic amplitude reduction normalized, besides the amplitudes of the primary cortical component consistently increasing with respect to HV and MOii.

In migraine, a strong relationship between clinical and neurophysiological profiles has been demonstrated previously. In episodic migraine, several studies have confirmed between attacks that the amplitude of sensory evoked potentials decreased or tended to be decreased for low numbers of delivered stimuli and lack of habituation during subsequent block averages [9,10,22]. Contrariwise, close to or during an attack, initial SSEP amplitude increased and deficit of habituation normalized [10,13,16] demonstrating that, on the one hand, the cortical activation state changes between the interictal and ictal periods, and that sensitization disappears between attacks, on the other hand. The electrocortical pattern we found here in our CM patients may thus suggest that the sensory cortex is persistently locked in an “ictal”-like state, associating with both sensitization and habituation. This pattern contrasts with that of episodic migraine where the transition between two electrocortical states (habituation and its lack) alternates following the recurrence of the migraine cycle (ictal and interictal). Similar results were recently observed in a visual EP study [11]. Moreover, this electrophysiological pattern partially differs from that found in chronic headache due to medication overuse (MOH) where, after a similar initial increase in response amplitude, a further increase was observed during stimuli repetitions [10]. Therefore, we considered MOH patients as locked in a “pre-ictal” state, in analogy with the cortical response pattern detected a few days before the beginning of an attack in episodic migraine [15,16]. Nevertheless, further underscoring the clinico-electrophysiological inter-relationship in migraine, this phenomenon in MOH evolved from migraine, was strongly dependent on the drug of overuse, since it was maximal in patients overusing NSAIDs and almost non-existent in those who overuse only triptans [10].

From a behavioural point of view, two distinct and independent processes govern the outcome of repetitive stereotyped sensory stimulation: an increasing one called sensitization, and a reducing one called habituation [24,25]. Sensitization occurs at the beginning of the test session and is responsible for the transitory increase in response amplitude, whereas habituation occurs throughout the test session, and is responsible for the late response decrement [26]. From a physiological point of view, demonstrative of central sensitization are the plastic changes in neural networks devoted to the processing of pain information [27] that results in abnormal neural excitability with decreased nociceptive thresholds and increased responsiveness to noxious and usually innocuous peripheral stimuli, such as, in our case, somatosensory ones [28]. Experimental studies in animals [29] and humans [30] show that SSEP amplitudes increase when transient intense activation of nociceptive afferents induces central sensitization, as happens in clinical pain conditions including chronic headache. Our study shows that sensitization, as reflected by increased initial SSEP amplitudes, is equally present in CM and MOi, although we managed not to record CM patients during a full-blown migraine attack. In CM and episodic migraine attacks central sensitization is associated with increased excitability both at the trigeminal system [31,32] and the supraspinal levels [33,34]. Interestingly, from our correlation analysis it emerges that the more pronounced the habituation, the more the CM number of headache days and sensory sensitization increase. This further reinforces the evidence for a strong relationship between clinical and neurophysiological features in migraine. A well-recognized clinical expression of central sensitization is cutaneous allodynia, which was shown to be prevalent during episodic migraine attacks at cephalic and extracephalic sites [35,36]. This phenomenon is even more evident in chronic migraine [37-39]. Based on animal models of experimental pain [40,41], some hypothesized that temporary sensitization of third-order thalamic neurons receiving convergent input from the dura, periorbital skin, and skin areas at different body sites explains the spread of cutaneous allodynia beyond the initial pain area during an attack of migraine [36,42]. In a recent study, Burstein and colleagues (2010) studied the effects of sensitizing skin stimuli on the activity of third-order trigeminovascular neurons in the rat thalamus, and on thalamic activation registered by fMRI during migraine in humans. On the one hand, the rat thalamus exhibited long-lasting hyperexcitability to cephalic and extracephalic skin stimuli in response to sensitizing inflammatory soup, and patients undergoing migraine experienced acute thalamic activation during the fMRI in response to extracephalic brush and heat stimuli, on the other hand [42]. The latter data indicate that allodynia is associated with sensitization of third-order thalamic neurons. Taking into account the latter evidence, our finding that the migraine attack modifies a neuronal activity (early HFO) that is generated in the thalamus is not surprising. We observed in both CM and episodic migraineurs recorded ictally an amplitude increase of the usually low thalamo-cortical activation (early HFO) of the interictal episodic migraineurs in parallel with a rise in primary cortical activation (late HFO). These findings may contribute to shed light on the mechanisms of central sensitization, since the elusive mechanisms that are able to ignite the cascade of events that finally lead to an attack facilitate the thalamic activity by reducing latency and augmenting amplitude and, in turn, enhance primary cortical activation at the low (1st SSEP amplitude block) and high frequency bands (late HFOs). This interpretation is supported by the direct correlation we found between the amplitude of the early and that of the late HFO components: in other words, the more the amplitude of the thalamic component increased, the more the cortical activation was enhanced, especially in CM and MOi patients. Furthermore, from the Pearson’s analysis it also emerged that the more this sequential thalamic and cortical HF oscillatory activation increased, the more marked the sensitization of the LF-SSEP N20-P25 1st amplitude block.

Only hypotheses can be made about the neural mechanisms by which thalamic neurons become facilitated in CM and in MOi. This process may primarily involve sequential sensitization of first-order or second-order trigeminovascular neurons probably through an evident (aura) or silent (without aura) cortical spreading depression or, more likely, through an indirect activation of pain modulatory structures in the brainstem (raphe magnus, locus coeruleus and other aminergic nuclei) and the forebrain (periaqueductal gray, rostroventral medulla) [43,44]. That the brainstem plays a relevant role in mediating and maintaining central sensitization was recently confirmed by functional neuroimaging studies in healthy humans [45,46]. In Migraine, clear examples of monoaminergic brainstem activation come from both neurophysiology [12,47] and neuroimaging studies where the dorsal rostral brainstem, which contains state-setting aminergic, including serotonergic, nuclei projecting to the thalamus and cortex, was activated immediately before [48] and during an attack of episodic [49,50] and chronic [4,51] migraine.

## Conclusions

In conclusion, we show for the first time that not only is the response pattern of the somatosensory cortex in CM patients similar to that found during a migraine attack in episodic migraine patients, in such a way that both habituate normally, as previously observed with visual responses [52], but also that they show an initial response sensitization. Moreover, from the analysis of high-frequency oscillations, it clearly emerges that this sensory sensitization may be explained by the fact that in both groups, the connections between the thalamus and cortex intensify compared to episodic migraine between attacks. Whether this electro-functional behaviour is primary or secondary to daily headache, thus reflecting an electrophysiological fingerprint of the somatosensory system central sensitization process remains to be determined.

## Abbreviations

CM: Chronic Migraine; HFOs: High-Frequency Oscillations; HV: Healthy Volunteer; LF: Low-Frequency; MOii: Migraineur without aura recorded interictally; MOi: Migraineur without aura recorded ictally; SSEP: Somatosensory Evoked Potential.

## Competing interests

The authors declare that they have no competing interests.

## Authors’ contributions

GC and EI made substantial contributions to interpretation of data as well as in drafting the manuscript. MS, CDL and FP were implied in the interpretation of data as well as in drafting the manuscript; MB and EI were implied in recording and analyzing data. All authors read and approved the final manuscript.
